# Associations of Parental Influences with Physical Activity and Screen Time among Young Children: A Systematic Review

**DOI:** 10.1155/2015/546925

**Published:** 2015-03-19

**Authors:** Huilan Xu, Li Ming Wen, Chris Rissel

**Affiliations:** ^1^Sydney School of Public Health, University of Sydney, Sydney, NSW 2006, Australia; ^2^Health Promotion Service, Sydney Local Health District, Level 9, King George V Building, Missenden Road, Camperdown, NSW 2050, Australia; ^3^Shanghai 10th People's Hospital, University of Tongji, Shanghai, China

## Abstract

Parents play a critical role in developing and shaping their children's physical activity (PA) and sedentary behaviours, particularly in the early years of life. The aim of this systematic review is to identify current literature investigating associations of parental influences with both PA and screen time in young children. This systematic review was conducted in November 2013 using 6 electronic databases covering research literature from January 1998 to November 2013. Thirty articles that met inclusion criteria were identified. These studies covered five important aspects of parenting: (1) parenting practices; (2) parents' role modelling; (3) parental perceptions of children's PA and screen viewing behaviours; (4) parental self-efficacy; and (5) general parenting style. Findings suggest that parents' encouragement and support can increase children's PA, and reducing parents' own screen time can lead to decreased child screen time. Improving parenting practices, parental self-efficacy or changing parenting style may also be promising approaches to increasing PA time and decreasing screen time of young children.

## 1. Introduction

Physical activity (PA) and sedentary behaviours (predominantly screen time) impact on the weight status of children [1–3]. Research evidence suggests that helping young children establish an active lifestyle can prevent them from overweight and obesity [4–7]. In the early years of life, parents play a critical role in developing and shaping their children's PA and sedentary behaviours through role modelling and creating a healthy home environment that increases PA and reduces screen time [8].

There have been several systematic reviews investigating correlates of PA and sedentary behaviours in young children [9–13]. These reviews used a social-ecologic framework [14] to summarize the correlates of PA and screen time of young children in five domains: (1) demographic and biological; (2) psychological, cognitive, and emotional; (3) behavioural attributes and skills; (4) social and cultural; and (5) physical environmental. Although parents' role modelling, parenting practices and parental perceptions of children's PA, and sedentary behaviours are part of the social and cultural domain, they have not been explicitly discussed in these reviews. General parenting style and parental self-efficacy that are also part of the social and cultural domain have not been investigated in these reviews.

In addition to parents' role modelling and parenting practices, general parenting style and parental self-efficacy also influence the development of PA and sedentary behaviours of young children, particularly in the early years of life [8]. Parenting style refers to a general pattern of parenting that provides the emotional background in which parents' behaviours are expressed and interpreted by a child [8]. It can be conceptualized as a context that moderates the influence of specific parenting practices on a child. Closely related to parenting practices and parenting style, parental self-efficacy is regarded as a parent's belief that he or she is capable of organizing and executing tasks related to parenting a child [15].

Findings from a recent systematic review [16] investigating associations between parental factors (parents' role modelling, parenting practices, and parental perceptions of importance and value of PA) and young children's PA were inconsistent, reporting that parental and family dynamics associated with children's PA are undeveloped. In particular, the review did not examine the parental influences on children's screen time (the time spent on watching TV, DVDs, or videos, using a computer and playing with an electronic game system) despite increasing research interest in investigating children's screen time and its independent association with childhood obesity. Further investigation was called for to clarify and understand specific parental influences that are associated with PA in children using comprehensive reviews of well-defined parental influences and their effects on both PA and screen time.

To fill in the knowledge gaps in this area, we aimed to update the current literature investigating parental influences and their associations with both PA and screen time in young children.

## 2. Methods

### 2.1. Search Strategy

In November 2013, a systematic search of the literature was conducted. Literature included in this review was retrieved from six electronic databases, including Medline, the Cochrane Central Register of Controlled Trials (CENTRAL), Cochrane Database for Systematic Review (CDSR), PsycINFO, EMBASE, and Web of knowledge. Research papers were limited to those written in English and published or included in databases from 1998 to November 2013. There was no restriction of study designs. Study participants in searched papers were limited to parents, father, and/or mother with young children aged ≤6 years. Parental influences included (1) parenting practices, (2) parents' role modelling (parents' own PA and screen time), (3) parental perceptions of children's PA and screen viewing behaviours, (4) parental self-efficacy, and (5) general parenting style. Children's PA included parent-reported “outdoor play” or “active play” and objectively measured PA level (e.g., accelerometer). The search strategy used for the Medline database is displayed in Table 1. A similar search strategy was used for other databases. Additional manual searches of the references of selected articles were also conducted for other relevant articles. Grey literature, such as unpublished studies and dissertations, was also included.

### 2.2. Study Selection

Study selection was based on predefined inclusion and exclusion criteria. The inclusion criteria were (1) individual quantitative studies that examined relationships of parental influences (covering at least one of the five aspects) with PA or screen time of young children; (2) studies with children aged ≤6 years old, or studies with a wide age range but describing the results specifically for children aged ≤6 years old; and (3) full text articles or dissertations, written in English. Studies were excluded from this review, if they were (1) pilot studies, (2) validation studies, (3) qualitative studies, (4) review papers, (5) studies that examined correlates of children's PA or screen time but did not include any aspects of defined parental influences for this review, and (6) studies involving children aged >6 years.

**Figure 1:**
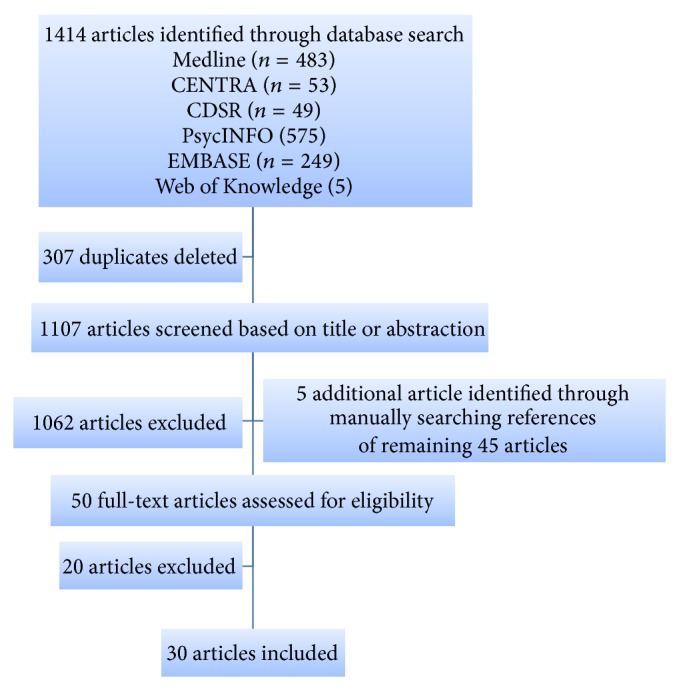
Flow diagram of study selection.

A total of 1414 articles were identified through database searching. Duplicate articles (*n* = 307) were removed, resulting in 1107 individual articles for consideration. By screening the titles, 1062 articles were considered to be irrelevant and thus excluded. Forty-five papers including grey literature remained as a result of the initial search. The references of these remaining 45 articles were further screened manually to identify other relevant articles. Five additional articles were included. A total of 50 full texts were further assessed. After excluding 20 articles according to the criteria, 30 articles were included in the present review. The process of study selection is reported in Figure 1.

### 2.3. Assessment of Included Articles

Two reviewers (Huilan Xu and Li Ming Wen) independently screened the study titles and abstracts and then critically appraised the selected articles. Due to heterogeneity of these studies (i.e., differences in study design, study quality, and statistical analysis method), it was not possible to conduct a meta-analysis that uses statistical methods to summarize the results. Therefore, the results from this review are presented descriptively. We critically evaluated the papers using a previously established quality checklist [17] with some modifications. This quality assessment tool was originally adapted from the Strengthening the Reporting of Observational Studies in Epidemiology (STROBE) statement [18]. For this review, the quality checklist consists of eight query items as follows: (1) was the study longitudinal or randomised controlled trail (RCT)? (2) Did the study describe the participant eligibility criteria? (3) Were the study participants randomly selected (or representative of the study population)? (4) Did the study report the sources and details of assessment for parental influences and did the instruments have acceptable reliability? (5) Did the study report the sources and details of assessments for PA and screen time and did all the methods have acceptable reliability for specific age group? (6) Did the study report a power calculation and was the study adequately powered to detect hypothesized relationships? (7) Did the study report the numbers of individuals who completed each of the different measures and did participants complete at least 80% of measures? (8) Did statistical analysis take into account the confounding? A score of “1” was assigned to “yes” to the query item, or a score of “0” was assigned. The range of score was from “0” to “8” for each paper. A paper with a score above 5 was regarded as a good quality paper.

## 3. Results

### 3.1. Description of Studies

Of the 30 articles included in this review, 14 studies were conducted in the United States, 6 in Australia, three in Canada, two in New Zealand, and one in each of Turkey, Greece, or Netherlands (Tables 2, 3, and 4). Fourteen studies [19–32] (Table 2) examined the association between parental influences and PA, with 12 studies [33–44] (Table 3) examined the association between parental influences and screen time, and four studies [45–48] (Table 4) examined associations of parental influences with both PA and screen time.

The quality scores of the papers ranged from 2 to 7 with an average score of 4.9. Most studies (*n* = 24) used cross-sectional design, with only 6 longitudinal studies having follow-up duration from 1 to 5 years. Sample sizes of these studies were reasonable based on their effect size and significance level except two studies had less than 100 participants.

### 3.2. Aspects and Measurement of the Parental Influences

#### 3.2.1. Parenting Practices

The review found that various components of parenting practices regarding PA [20–23, 25–29, 31, 45, 46] or screen time [33–35, 38, 43, 45, 46, 48] were reported by 20 included studies. For child PA, parenting practices included (1) parents encouragement or support (e.g., parents participated in PA with their child, provided transportation to PA facilities, watched the child in activities, or let their child know that PA is good for health); (2) setting rules (e.g., restricting indoor games and having TV viewing rules); (3) parents preference (e.g., preferring child to do the same activities with their older siblings). For children's screen time, parenting practices included (1) setting TV rules (e.g., time or program rules); (2) watching TV with their child; (3) monitoring screen time; (4) having meals without watching TV; and (5) stimulating children to be active.

#### 3.2.2. Parents' Role Modelling (Parents' Own PA and TV/Screen Time)

Eight studies [19, 21–24, 28, 29, 48] used parent self-administered questionnaires to assess parents' own PA time, frequency, and intensity with two studies using accelerometer [20, 30] and one study using both parent self-administered questionnaire and accelerometer [32]. Nine studies used a questionnaire to measure parental TV time [33–35, 37–40, 42, 44]. Of them, two studies measured screen time rather than TV time [34, 37].

#### 3.2.3. Parental Perceptions

Perception of importance of children's PA [23, 28] and perception of children's physical competence [21, 22] were reported in 4 studies. In examining children's screen time, perception of “too much screen time” [33, 38], “TV helps children's learning” [37, 43], or “TV hurts children's learning” [43, 46] were reported in five studies.

#### 3.2.4. Parenting Self-Efficacy

Parental self-efficacy for limiting screen time was assessed by a single item: the level of confidence that parent could say “No” to their child's request for screen time (TV/computer/video games) [36–38]. One study assessed parental self-efficacy for influencing their child's PA in eight challenging situations such that the parent cannot think of activities to suggest; the parent is not able to participate in the activity [47].

#### 3.2.5. Parenting Style

Only one study investigated the association of general parenting style and children's PA [31]. Parenting style was classified by using Maccoby and Martin's [49] classifications of parenting style with two dimensions - responsiveness (parental warmth/hostility) and demandingness (parental control).

### 3.3. Classification and Measurement of Children's PA and Screen Time

Children's PA was measured by parent self-administered questionnaire in 10 studies [19, 23, 24, 26, 27, 31, 45–48], followed by accelerometer in 7 studies [20–22, 28–30, 32], or heart rate monitoring and direct observation in one study [25].

Screen time was assessed by totaling the time spent on TV, DVDs, electronic game, and computer (*n* = 16 studies), which was always measured by parent self-administered questionnaire.

### 3.4. Parental Influences and Young Children's PA

Table 5 summarized the associations between parental influences and children's PA. Through examining three aspects of parenting practices on children's PA, there was moderate to strong evidence of linkage between parental encouragement/support and children's PA. For example, of 11 studies examining the relationship between parents' encouragement/support and children's PA, eight studies [21, 23, 26–29, 31, 45] with a mean quality score of 5.6 found that children whose parents encouraged or supported them to do PA were more likely to have higher levels of PA, yet three studies (with a mean quality score of 4.7) did not find such association [20, 22, 25]. The associations of setting rules and parental preference with children's PA were weak due to a small number of studies and inconsistent findings. One study (a quality score of 7) examining both setting PA rules and parent preference found that parental rules were positively associated with boy's PA, but parent preference was negatively associated with boy's PA [29]. Among three studies examining the association between setting TV viewing rules and children's PA, two studies (a mean quality score of 3.5) found that children with TV viewing rules spent more time playing outdoors [46, 48], which was not supported by Gubbels et al.'s study [45].

There was also moderate to strong evidence of positive association between parental PA level and young children's level of activity. Eight studies with a mean quality score of 5.1 found that parents' own PA level was positively associated with young children's PA [19, 20, 23, 28–30, 32, 48] with only two studies revealing no such association [22, 24].

Weak evidence was found in examining associations of parental perception, parental self-efficacy, and parenting style with children's PA due to limited studies and mixed findings. For example, contradictive findings were reported on parental perception of the importance of PA and children's PA in two studies [23, 28]. But another two studies found that parent perception of children's physical competence was positively associated with children's PA (MVPA) [21, 22]. Only one study found that parents who have a high sense of self-efficacy are more likely to have their children meeting the PA guidelines [47]. General parenting style was not found to be associated with child PA. However, parental warmth, one of the parenting style dimensions, was positively associated with child PA [31].

### 3.5. Parental Influences and Young Children's Screen Time

Table 6 summarized the associations between parental influences and children's screen time. Weak and mixed evidence was found in examining association between parenting practices and screen time. Of 7 studies examining TV time rules, four studies (a mean quality score of 5) found that setting TV time rules resulted in less screen time [33–35, 46] and two studies (a mean quality score of 4.5) found no such association [38, 43] with another study suggesting a negative effect of TV time rules on screen time [45]. Mixed evidence was found from two studies in examining setting TV program rules and screen time [43, 46]. The study conducted in 2005 found children whose parents had TV program rules watched more TV [46], but the finding was not supported by another study in 2007 [43]. In addition, one study revealed that more family rules about TV viewing were associated with less screen time [48]. Coviewing TV with a child [35, 46] and having meals when TV is on [34, 43] were associated with increased screen time. In addition, one study [45] found that monitoring child screen time was not associated with screen time, but stimulating a child to be active was associated to less screen time.

In contrast to parenting practices, the review found that there was moderate evidence suggesting parental self-efficacy and parents' own TV time were associated with children's screen time. Nine studies with a mean quality score of 4.8 consistently revealed that parents' own TV time was positively associated with their child's screen time [33–35, 37–40, 42, 44]. The evidence was also consistent in four studies (a mean quality score of 5.5), which concluded that high parental self-efficacy in reducing children's screen time was associated with less screen time in children [36–38, 47].

In terms of associations of parental perceptions and parenting style with screen time, the evidence was weak and inconsistent from only four studies reviewed. For example, two studies revealed that parental perception of their child spending too much time on playing video games or watching TV was associated with increased screen time [33, 38]. Parental perception of “TV helps” was found to be associated with increased screen time [37, 43]. Parental perception of “TV hurts” was associated with decreased screen time [46]. But this association was not supported by another study conducted by the same author [43]. The authoritative parenting style was found to be associated with decreased children's screen time by only one study [41].

## 4. Discussion

By critically assessing and synthesizing evidence from individual studies, the present systematic review updates the current literature and fills knowledge gaps in relation to associations of parental influences with young children's PA and screen viewing behaviours. Moderate to strong evidence was found in relation to the associations of parental encouragement/support for PA and parents' own PA level with children's PA. Moderate evidence was also found regarding associations of parental self-efficacy and parents' own screen time with children's screen time. Associations of other aspects of parenting practices, parental perceptions, and parenting style with children's PA and screen time were indeterminate due to limited studies and contradictory results from studies included in this review.

### 4.1. What Is Already Known?

Three previous systematic reviews summarised mixed evidence of associations of parents' PA and parental encouragement/support with children's PA [10, 12, 16]. Hinkley et al. and Mitchell et al. concluded that parents' PA was positively associated with young children's PA [12, 16], while de Craemer et al. found indeterminate association [10]. Likewise, Mitchell et al. found that there was positive association between parental encouragement/support and children's PA [16], yet Hinkley et al. and de Craemer et al. found no such association [10, 12]. Another systematic review that included several qualitative studies suggested that parent involvement, encouragement, and modelling of PA may be important influences impacting young children's PA and are worthy of further systematic study [50].

Of four previous systematic reviews of association between parents' TV time and children's screen time, two concluded a positive association [9, 11], while the other two found that there was a positive but indeterminate association [10, 13]. Mixed findings about the association between parental TV rules and children's screen time were reported by three reviews [9, 10, 13]. Cillero and Jago and Hinkley et al. found that children with TV rules had less screen time [9, 13], while de Craemer et al. found that this association was indeterminate [10].

Mixed evidence in relation to associations of parental influences with children's PA and screen time was further explored by some qualitative studies. For example, Dwyer et al. found that that parental modelling and/or encouragement of activity was a key influence and predictor of PA and sedentary behaviour in children, which was especially acknowledged by parents [51]. Another qualitative study found parents' own screen viewing habits was one of the two most frequently mentioned factors that influence children's screen time (the other one was weather conditions) [52]. Parents also liked the idea of implementing parental rules for TV viewing (e.g., time rules, no TV viewing during meals) [52].

### 4.2. What This Study Adds

The present review examines the evidence of associations of parental influences with both PA and screen time of young children. Findings from the review generally support and reinforce the evidence found in some of the previous reviews. This review also examined the role of parental rules for PA and screen viewing, parent perceptions of children's PA, and screen viewing and found their effects on children's PA and screen time remained indeterminate because of contradictive findings or a small number of studies.

Unlike previous reviews that only focused on parenting practices, the present review also explores parental self-efficacy and general parenting style as part of the parental influences. Moderate evidence was found that increased parental self-efficacy was associated with reduced children's screen time. But, the effect of parental self-efficacy on increasing children's PA cannot be concluded with only one study found in this review. The association of parenting style with children's PA and screen time remains unclear due to the limited number of studies.

### 4.3. Evidence Gaps and Future Research

Despite substantial evidence suggesting that an authoritative parenting style was associated with older children's PA and sedentary behaviours [53, 54], the present systematic review was not able to make such conclusion for young children with only one study included in the review [31, 41]. Therefore, the associations of parenting style and young children's PA and sedentary behaviours need more attention in future research. In this review, only one longitudinal study found that the relationship between parent's PA and preschool children's PA was stronger than that in older children (at follow-up) [32]. It seems likely that the associations of various parental influences and children's PA and screen viewing behaviours would change with advancing age of children. Hence, further longitudinal research on parental influences is needed. In addition, there is a clear evidence gap regarding the effect of parental self-efficacy on children's PA.

As discussed above, associations of some parenting practices (e.g., setting PA and TV rules) and parental perceptions with children's PA and screen time are still indeterminate and further investigation is needed to inform the development of health promotion programs. Inconsistent findings of the association between setting TV rules and children's screen time could be a result of different levels of obedience of rules. One qualitative study involving six European countries revealed that, in general, parents of preschool-school children only had informal rules about TV viewing [52]. In this review, two individual studies were conducted by Vandewater et al. in United States in 2005 and 2007 [43, 46]. The first study found that parental perceptions of “TV hurts” and setting TV time rules were associated with decreased screen time [46]. However, such associations were not found in a later study [43]. It may be that the association between parental perception of “TV hurts” and children's screen time was related to setting TV time rules. Thus, more research is needed to investigate whether the parental perception of TV “hurts” influences establishing and enforcing TV rules. In addition, whether parenting practices are moderated by general parenting style also needs to be investigated.

## 5. Conclusions

In the early years of life, some parental influences were significantly associated with young children's PA and screen time with moderate to strong evidence. Results from the present review suggest that parents' encouragement and support can increase their children's PA and reducing parents own screen time can lead to decreased child screen time. Improving parenting practices, parental self-efficacy and parenting style may also be promising approaches to increasing PA time and decreasing screen time of young children.

## Figures and Tables

**Table 1:** The search strategy used for Medline database.

Database: Ovid Medline(R) *〈*1946 to November Week 3 2013*〉* search strategy:	
(1) preschool^*^.mp. (763123)	
(2) young child^*^.mp. (34520)	
(3) early child^*^.mp. (15721)	
(4) toddler.mp. (1946)	
(5) (1) or (2) or (3) or (4) (783324)	
(6) parenting style^*^.mp. (762)	
(7) parenting practice.mp. (26)	
(8) parenting behavio^*^.mp. (847)	
(9) parenting.mp. (14946)	
(10) maternal influence^*^.mp. (460)	
(11) parental influence^*^.mp. (439)	
(12) parental self-efficacy.mp. (79)	
(13) parental confidence.mp. (51)	
(14) parental rules.mp. (61)	
(15) parental attitudes.mp. (730)	
(16) parental concerns.mp. (324)	
(17) parent^*^ support^*^.mp. (1197)	
(18) parent^*^ encouragement.mp. (80)	
(19) parent^*^ involvement.mp. (1051)	
(20) parent modeling.mp. (22)	
(21) (6) or (7) or (8) or (9) or (10) or (11) or (12) or (13) or (14) or (15) or (16) or (17) or (18) or (19) or (20) (18529)	
(22) physical activit^*^.mp. (55070)	
(23) total PA.mp. (264)	
(24) MVPA.mp. (1001)	
(25) PA.mp. (54822)	
(26) VPA.mp. (3623)	
(27) physical exercise.mp. (9183)	
(28) outdoor play.mp. (102)	
(29) active play.mp. (87)	
(30) play.mp. (449998)	
(31) leisure activit^*^.mp. (7741)	
(32) (22) or (23) or (24) or (25) or (26) or (27) or (28) or (29) or (30) or (31) (569321)	
(33) physical inactivity.mp. (3827)	
(34) sedentary behavio^*^.mp. (1660)	
(35) television viewing.mp. (957)	
(36) TV viewing time.mp. (96)	
(37) TV viewing.mp. (503)	
(38) TV time.mp. (71)	
(39) DVD^*^.mp. (951)	
(40) video viewing.mp. (83)	
(41) computer using.mp. (512)	
(42) computer time.mp. (255)	
(43) electronic game.mp. (34)	
(44) screen time.mp. (449)	
(45) small-screen recreation.mp. (15)	
(46) (33) or (34) or (35) or (36) or (37) or (38) or (39) or (40) or (41) or (42) or (43) or (44) or (45) (8478)	
(47) (32) or (46) (573869)	
(48) (5) and (21) and (47) (553)	
(49) limit (48) to (English language and humans and yr = “1998–current”) (483)	

^*^The asterisk sign stands for any character(s).

**Table 2:** Association between parental influence and children's PA.

Author (year) country reference	Study design	Sample	Age (years)	Parental influence (measurement)	Child PA (measurement)	Adjusted confounders	Main findings	Quality score
Alderman et al. (2010) USA [19]	Longitudinal	69	Baseline 4–6 Follow-up 5–15	(1) Parental PA (2) The amount of time parents report engaging in PA together with their child. (parent self-administered questionnaire)	Children's PA (parent self-administered questionnaire)	No	(1) At baseline, the relationship between parental and children's PA was moderately strong with Pearson's *r* = 0.44 (*P* < 0.05). (2) At follow-up, this relationship was weaker and no longer significant (Pearson's *r* = 0.08, *P* > 0.05). (3) The amount of time parents report engaging in PA together with their child decreased significantly over time.	4

Oliver et al. (2010) New Zealand [20]	Cross-sectional	93	2–5	(1) Parental PA rate (2) Parental PA supports/inhibitors Encouraged child to be active Parent was physically active with child Child was taken out to playground, park, beach, and so forth Restrict TV viewing	Children's PA rate (Actical accelerometers)	Children's age	**Univariable linear regression model** (1) Parental PA supports/inhibitors were not associated with children's PA rate. **Multivariable linear regression model** (2) After adjusting children's age, parental PA rate was positively associated with children's PA rate (*β* = 0.09, *P* = 0.01).	6

Loprinzi and Trost (2010) Australia [21]	Cross-sectional	156	2–5	(1) Parental PA (2) Parental enjoyment of PA (3) Parental support for PA (4) parental perceived importance of child PA (5) Parents' perceptions of their children's physical competence (parent self-administered questionnaire)	(1) Children's PA at home (parent self-administered questionnaire, PAEC-Q) (2) Children's PA at child care (Actigraph 7164 acclerometer)	(1) Children's age (2) Child gender	**Path analysis** (1) Parental PA and parents' perceptions of their children's physical competence were positively associated with parental support for PA (*β* = 0.23 and 0.18, respectively, *P* < 0.05). (2) Parental enjoyment of PA and perceived importance of child PA were not significantly associated with parental support for PA. **Home-based children's PA** (3) Parental support for PA was positively associated with home-based children's PA (*β* = 0.16, *P* < 0.05). (4) Parents' perceptions of their children's physical competence were directly and positively associated with home-based children's PA (*β* = 0.2, *P* < 0.05). **Child care MVPA** (5) Parental support for PA was not associated with children's MVPA at child care (*β* = 0.01, *P* = 0.94). (6) Parents' perceptions of their children's physical competence were directly and positively associated with children's MVPA at child care (*β* = 0.28, *P* < 0.001).	5

Pfeiffer et al. (2009) USA [22]	Clustered cross-sectional	331	3–5	(1) Parental VPA (2) Parental perception of their child's athletic competence (3) Family support for PA (average of responses to frequency of following items) Family encouraged PA, participated in PA with child, provided transportation to PA facilities, watched the child in activities, and told the child that PA is good (parent self-administered questionnaire)	Children's MVPA and NSA (ActiGraph accelerometers)	(1) Child gender (2) Child race (3) Child BMI *z*-score (4) Parent education	**Mixed model** (1) Parental perception of their child's athletic competence was positively associated with children's MVPA and NSA (*β* = 0.39 and 0.80, respectively, *P* < 0.01). (2) Parental VPA and family support for PA were not significantly associated with children's MVPA and NSA.	5

Zecevic et al. (2010) Canada [23]	Cross-sectional	102	3–5	(1) Parental PA (2) Family support for PA (average of responses to frequency of following items) Family encouraged PA, participated in PA with child, provided transportation to PA facilities, watched the child in activities, and told the child that PA is good (3) Parents' enjoyment of PA (4) Importance of child's PA ability (parent self-administered questionnaire)	(1) Children's PA (active play/sports) (2) Parent's perceived intensity of a child's PA (parent self-administered questionnaire)	Not reported	**Multiple logistic regression model** **Children's PA** (1) The greater parents' enjoyment of PA is, the more likely the children were to engage in the recommended amount of daily PA (PA ≥1 hour/day) (AOR 2.01, *P* < 0.05). (2) Parental PA and family support for PA were positively associated with children's PA (AOR 1.62 and 2.18, respectively, *P* < 0.10). (3) Importance of child's PA ability was not associated with children's PA. **Intensity of children's PA** (4) Parental PA and family support for PA were positively associated with children's intensity of PA (AOR 1.97 and 4.22, respectively, *P* < 0.05). (5) Parents' enjoyment of PA and importance of child's PA ability were not associated with children's PA.	3

Davison and Birch (2001) USA [24]	Longitudinal	185	Baseline 5 Follow-up 7	Parental PA (parent self-administered questionnaire: the level of PA: low, medium, and high)	Girl's PA (level of active) (parent self-administered questionnaire: the level of PA: low, medium, and high)	No	No associations between girls' and parents' total PA at 5 years of age.	4

Jago et al. (2005) USA [25]	Longitudinal	149	Baseline 3-4 3-year follow-up	Parental encouragement/discouragement for PA (direct observation)	(1) Children's PA (1) Heat rate monitoring (2) Direct observation with Children's Activity Rating Scale)	(1) Child BMI (2) Child ethnicity (3) Child gender	**In year 2 of the follow-up** (1) Observed children's PA was positively associated with encouragements (*r* = 0.19, *P* < 0.05) and discouragements (*r* = 0.349, *P* < 0.01) for PA. **In year 3 of the follow-up** (2) Observed children's PA was significantly associated with encouragements (*r* = 0.381, *P* < 0.01) and discouragements (*r* = 0.418, *P* < 0.01) for PA. (3) Encouragements and discouragements of PA by parents were not significant predictors of PA in regression models.	3

Beets and Foley (2008) USA [26]	Clustered cross-sectional	10694	5-6	(1) Father-child time (2) Family sports/activities together time (parent self-administered questionnaire)	Child physical activity (parent self-administered questionnaire)	(1) Child race (2) Child gender (3) Child BMI	(1) Father-child time was positively associated with child PA on weekdays (*β* = 0.09, *P* < 0.001) and weekends (*β* = 0.07, *P* = 0.002). (2) Family sports time was positively associated with child PA (*β* = 0.27, *P* < 0.05). (3) The effect of father-child time on child PA was mediated by family sports time. The indirect effect of father-child time on child PA was 0.03, *P* < 0.001 on weekdays, and 0.02, *P* < 0.001 on weekends.	5

Cleland et al. (2010) Australia [27]	Longitudinal	130	Baseline: 5-6 5-year follow-up	Parental encouragement of playing outdoors (parent self-administered questionnaire)	Child time spent outdoors (parent self-administered questionnaire)	(1) Maternal education (2) Parental marital status (3) Other	Compared to low parental encouragement, high parental encouragement was associated with more time spent outdoors on average of over 5 years for girls (234 minutes/week, 95% CI 30.1–437.8), but not for boys.	7

Dowda et al. (2011) USA [28]	Cross-sectional	411	3–5	(1) Family support Encourage child PA Play with child outdoors Provide transportation Watch child do PA or play outdoors games Tell child PA is good (2) Parent PA (3) Parent enjoys PA (4) Importance of child PA (parent self-administered questionnaire)	Child PA (MVPA created from (1) ActiGraph accelerometers (2) Direct observation (3) Parent-reported child's athletic coordination)	(1) Child gender (2) Preschool quality (3) Home PA equipment	(1) Family support was directly and significantly associated with child MVPA (*β* = 0.28). (2) Parent PA was indirectly associated with child's MVPA (*β* = 0.015, 95% CI 0.01–0.036) (mediated by family support). (3) Parent enjoys PA that was indirectly associated with child's MVPA (*β* = 0.031, 95% CI 0.005–0.065) (mediated by family support). (4) Importance of child participation in PA was indirectly associated with child's MVPA (*β* = 0.014, 95% CI 0.001–0.034) (mediated by family support).	6

Hinkley et al. (2012) Australia [29]	Clustered cross-sectional	705 (weekly) 773 (weekday) 605 (weekend)	3–5	(1) Father/mother PA (2) PA interaction (3) Parent housework (4) Parent preference (child do the same activities as older siblings) (5) Parental rules restricting rough games inside (6) Parental logistic support (parent self-administered questionnaire)	Child PA (1) Weekly (2) Weekday (3) Weekend-day (ActiGraph accelerometers)	Child age Child daily sleep Number of siblings Other variables showed significance in models	**Boys:** (1) Mother's PA interaction with child was positively associated with boy's weekly PA (AOR 1.01, 95% CI 1.00–1.03). (2) Housework was negatively associated with boy's weekday PA (AOR 0.91, 95% CI 0.85–0.97). (3) Parent prefers child to do same activities as older children were negatively associated with boy's weekly (AOR 0.94, 95% CI 0.88–0.99) and weekday PA (AOR 0.92, 95% CI 0.87–0.97). (4) Rules restricting rough games inside were positively associated with boy's weekly PA (AOR 1.06, 95% CI 1.01–1.12) and weekend-day PA (AOR 1.11, 95% CI 1.02–1.20). **Girls:** (5) Paternal time in moderate PA (not total PA) was significantly associated with girl's weekly PA (AOR 1.01, 95% CI 1.00–1.02). Maternal time in vigorous PA was not significantly associated with girl's weekly, weekday, and weekend-day PA. (6) Paternal provision of logistic support was significantly associated with girl's weekend-day PA (AOR 1.03, 95% CI 1.00–1.05).	7

Ruiz et al. (2011) USA [30]	Cross-sectional	106 (80)	3–5	(1) Parent time spent in sedentary behaviour (2) Parent PA (light, moderate, and vigorous) (ActiGraph accelerometers)	(1) Child time spent in sedentary behaviour (2) Child PA (light, moderate, and vigorous) (ActiGraph accelerometers)	None for Pearson's correlation. Child age and gender for linear regression	(1) There was a strong and positive correlation between parents and their children's daily sedentary (Pearson's *r* = 0.597, *P* < 0.001), mild (Pearson's *r* = 0.895, *P* < 0.0001), and moderate (Pearson's *r* = 0.739, *P* < 0.0001) PA levels but not for vigorous PA levels (Pearson's *r* = −0.07, *P* = 0.56). (2) Parent PA was significantly associated with child's PA (*β* was not reported, *P* < 0.0001).	6

Schary et al. (2012) USA [31]	Cross-sectional	195	2–5	(1) Parental support Encouragement Playing with the child Providing transportation Watching the child participate in PA Providing information about PA (2) Dimensions of parenting style Warmth Control Irritability (3) Parenting style Authoritative Authoritarian Permissive Neglectful (parent self-administered questionnaire)	Active play (parent self-administered questionnaire with PAEC-Q)	Parent gender	(1) Parental support was positively associated with child active play (*β* = 0.30, *P* < 0.001). (2) Parental warmth was positively associated with child active play (*β* = 0.15, *P* = 0.04). Parental control and irritability were not significantly associated with child active play. (3) Parenting style was not associated with child active play. (4) After adjusted for parenting style, parental support remained positively associated with child active play (*β* = 0.30, *P* < 0.001). (5) Parenting style did not moderate the relationship between parental support and child active play behaviour.	6

Taylor et al. (2009) New Zealand [32]	Longitudinal	244	Baseline: 3 Follow-up at 4 and 5	(1) Mother's PA (2) Father's PA ((a) Actical accelerometers (b) Parent self-administered questionnaire)	Child PA ((1) Actical accelerometers. (2) Parent self-administered questionnaire)	(1) Child age (2) Child sex (3) Days of the week (week day/weekend day)	**Cross-sectional analysis:** (1) Parental activity was weakly associated with child's PA measured by AAC at 3 and 4 years but not 5 years (Spearman rank-order *r* = 0.08, *P* = 0.034 and *r* = 0.17, *P* = 0.051 for mothers and *r* = 0.28, *P* = 0.001 and *r* = 0.23, *P* = 0.007 for fathers for 3 and 4 years, respectively). (2) Mother's PA was associated with child's PA by parental ratings at 4 and 5 years but not 3 years (*r* = 0.21, *P* = 0.003 and *r* = 0.18, *P* = 0.014). (3) There was no association between parental ratings of their own and their child's PA. **Mixed model:** (4) Father's PA was a significant predictor of the child's PA measured by AAC (adjusted *β* = 0.11, *P* = 0.024). (5) Mother's PA was not significantly associated with child's PA measured by AAC (adjusted *β* = 0.21, *P* = 0.062).	6

MVPA: moderate to vigorous physical activity. PAEC-Q: Physical Activity and Exercise Questionnaire for Children. VPA: vigorous physical activity. NSA: nonsedentary activity. AOR: adjusted odds ratio. AAC: average accelerometry counts.

**Table 3:** Association between parental influence and children's screen time.

Author (year) country reference	Study design	Sample	Age (years)	Parental influence (measurement)	Child PA (measurement)	Adjusted confounders	Main findings	Quality score
Barr- Anderson et al. (2011) USA [33]	Cross-sectional	431	Mean age 5.8 SD 0.51	(1) Parent TV time (2) Parent limit child's TV (3) Parental perception of child's screen time (parent self-administered questionnaire)	Child screen time (parent self-administered questionnaire)	(1) Intervention condition (2) Child age (3) Parent BMI (4) Relative socioeconomic status	(1) Parental TV viewing time was positively associated with child screen time (*β* = 0.37, *P* < 0.001). (2) Parent often or always limits child's TV was associated with less child screen time (*β* = −0.38, *P* = 0.01). (3) Parental perception that the child spent too much time playing video games was positively associated with child screen time (*β* = 1.06, *P* < 0.001).	6

Birken et al. (2011) Canada [34]	Cross-sectional	157	3	(1) Parent screen time (2) Screen time rules (3) Meals with the TV on (parent self-administered questionnaire)	Child screen time (parent self-administered questionnaire)	(1) Maternal education (2) Maternal age	(1) An increase of 1 hour of parental screen time was associated with 12 (95% CI 6–18) minutes of increase per day of child weekend screen time. (2) Screen time rules decreased child weekend screen time by 30 (95% CI 6–54) minutes per day. (3) Eating lunch in front of the screen was associated with 78 (95% CI 36–132) minutes of increase per day of child weekday screen time and 96 (95% CI 30–192) minutes of increase per day of child weekend-day screen time.	5

Bleakley et al. (2013) USA [35]	Cross-sectional	465	≤5	(1) Parent TV time (2) Coviewing TV with child (3) TV time restriction (parent self-reported online survey)	Child TV viewing time (parent self-reported online survey)	(1) Parental well-being (2) Media access TV in bedroom Number of TV sets PC in bedroom (3) Demographic Child gender Child age Parent race Parent education Parent income Parent employment Number of children	(1) Parent TV viewing was significantly associated with child TV time (*β* = 0.47, *P* < 0.05). (2) Coviewing TV with child was positively associated with more child TV time (*β* = 0.16, *P* < 0.05) (3) Parent TV time restriction was associated with lower child TV time.	5

Campbell et al. (2010) Australia [36]	Cross-sectional	140	1 (*n* = 60) 5 (*n* = 80)	(1) Maternal self-efficacy to promote PA to displace TV viewing (2) Maternal self-efficacy to limit TV viewing (parent self-administered questionnaire)	Children's TV (TV, DVD, and video) time (parent self-administered questionnaire)	No	(1) Maternal self-efficacy to promote PA to displace TV viewing was significantly inversely associated with both groups of children's TV time (Spearman rank order correlation −0.28 and −0.27, *P* < 0.05 for 1-year and 5-year-old children, respectively). (2) Maternal self-efficacy to limit TV viewing was significantly inversely associated with both groups of children's total TV time (Spearman rank order correlation −0.38, *P* < 0.005, and −0.31, *P* < 0.05 = 1 for 1-year- and 5-year-old children, respectively). (3) Mothers of 1-year-old children indicated significantly higher self-efficacy for limiting TV viewing than mothers of 5-year-old children (*P* < 0.005).	4

Carson and Janssen (2012) Canada [37]	Cross-sectional	746	≤5	(1) Parents' screen time (2) Parents' self-efficacy in reducing children's screen time (3) Parent attitudes regarding children's screen time (4) Parents' barriers to reduce children's screen time (5) Parents' perception of typical screen time in children (parent self-administered questionnaire)	Child screen time (parent self-administered questionnaire)	Sequential linear regression models adjusted for (1) Child age (2) Siblings (3) Education (4) Income (5) Family structure (6) Parental cognitions Multiple logistic regress models adjusted for (7) Child age (8) Child gender (9) Income (10) Parental cognitions (11) TV in bedroom (12) Video games in bedroom	**Sequential linear regression models** (1) Parents' screen time was positively associated with children's screen time (*β* = 0.13, 95% CI 0.07 to 0.19). (2) Parents' self-efficacy was negatively associated with children's screen time (*β* = −0.38, 95% CI −0.64 to −0.11). (3) Parents' attitude was positively associated with children's screen time (*β* = 1.68, 95% CI 1.19 to 2.17). (4) Parents' barrier was positively associated with children's screen time (*β* = 0.99, 95% CI 0.57 to 1.42). (5) Parents' perception of typical screen time in children was positively associated with children's screen time (*β* = 1.16, 95% CI 0.97 to 1.35). When separated children by 0–3 yr olds and 4-5 yr olds, parents' screen time and self-efficacy were associated with children's screen time for 4-5 yr olds, but not for 0–3 yr olds. **Multiple logistic regress models** (1) Parents' screen time was associated with children's high (top quartile) screen time (AOR 1.76, 95% CI 1.43–2.17). (2) Parents' self-efficacy was negatively associated with children's high (top quartile) screen time (AOR 0.62, 95% CI 0.49–0.78). (3) Parents' attitude was positively associated with children's high (top quartile) screen time (AOR 1.48, 95% CI 1.12 to 1.91). (4) Parents' barrier was not associated with children's high (top quartile) screen time. (5) Parents' perception of typical screen time in children was positively associated with children's high (top quartile) screen time (AOR 2.78, 95% CI 2.21 to 3.51).	6

Hinkley et al. (2013) Australia [38]	Clustered cross-sectional	935	Mean age 4.54 SD 0.70	(1) Maternal TV time (2) Paternal TV time (3) Parental concern about child screen time (4) Parent prefers child to do activities older children do (5) Parent believes the amount of TV child watches would not affect child health (6) Parental confidence to say no to child requests to play video games (7) Parent limits child TV view (parent self-administered questionnaire)	Child screen time (parent self-administered questionnaire)	(1) Child age (2) Child quiet play time (3) Maternal education (4) Physical environmental variables	**Boys** (1) Maternal TV time but not paternal TV time was negatively associated with boys meeting Australian/Canadian screen time recommendations (<1 hour/day) (AOR 0.90, 95% CI 0.84–0.97). (2) Boys were more likely to meet Australian/Canadian screen time recommendations if their parents had the confidence to say no to their child's requests to play video games (AOR 3.14, 95% CI 1.41–7.00). (3) Parent limits child TV viewing was not significantly associated with boys meeting Australian/Canadian screen time recommendations. **Girls** (1) Maternal TV time but not paternal TV time was negatively associated with girls meeting Australian/Canadian screen time recommendations (AOR 0.87, 95% CI 0.80–0.94). (2) Girls were less likely to meet Australian/Canadian screen time recommendations if their parents are concerned about their screen time (AOR 0.81, 95% CI 0.72–0.91). (3) Girls were less likely to meet Australian/Canadian screen time recommendations if their parent prefers child to do activities older children do (AOR 0.36, 95% CI 0.15–0.82). (4) Parent believes the amount of TV child watches would not affect child health was not significantly associated with girls meeting Australian/Canadian screen time recommendations. (5) Parent limits child TV viewing was not significantly associated with girls meeting Australian/Canadian screen time recommendations.	6

Kourlaba et al. (2009) Greece [39]	Clustered cross-sectional	2374	1–5	(1) Mother TV viewing time (2) Father TV viewing time (parent self-administered questionnaire)	Child TV viewing time (parent self-administered questionnaire)	Child: (1) Gender (2) PA Parent: (3) Educational status (4) Maternal employment (5) Time parents spent with children (6) Siblings	(1) In the total sample, parental TV time was significantly and positively associated with child TV time. (2) Mother TV time was significantly associated with TV time (≥2 h/day) of children aged 3–5 years (AOR 1.38, 95% CI 1.25–1.57) but marginally associated with children aged 1-2 years (AOR 1.24, 95% CI 0.94–1.79). (3) Father TV time was significantly associated with TV time (≥2 h/day) of children aged 3–5 years (AOR 1.24, 95% CI 1.15–1.42) but not children aged 1-2 years.	5

Rideout et al. (2006) USA [40]	Cross-sectional	1051	6 months–6 years	Parent TV time (parent self-reported telephone survey)	Children's TV time (parent self-reported telephone survey)	None	(1) Children whose parents watched TV ≥2 hrs/day watched averaging 17 minutes more TV per day than children whose parents spend 1-2 hrs/day (74 minutes versus 57 minutes, *P* < 0.005), and 28 minutes more TV per day than children whose parents spend <1 h/day (74 minutes versus 46 minutes, *P* < 0.005).	2

Schary et al. (2012) USA [41]	Cross-sectional	201	2–5	Parenting style (parent self-administered Child Rearing Questionnaire)	(1) Screen time (2) Quiet play time (parent self-administered questionnaire)	Child age Parent BMI	(1) Compared to neglectful parenting style, authoritative parenting style was associated with less child screen time on weekend (*β* = −0.17, *P* = 0.05 with adjusted model; *β* = −0.26, *P* = 0.002 with unadjusted model) and weekday (*β* = −0.21, *P* = 0.01 with unadjusted model). (2) Parenting style was not associated with child quiet play time.	6

Thompson et al. (2013) USA [42]	Longitudinal	217	Baseline: 3 months Follow-up at 6, 9, 12, and 18 months	Mother TV viewing time (parent self-administered questionnaire)	Infant TV exposure time (time spend in front of the TV) (parent self-administered questionnaire)	(1) Child gender (2) Visit	Mother TV viewing time was positively associated with infant TV exposure time (≥1 h/day) with AOR 1.27 (95% CI 1.12–1.44).	5

Vandewater et al. (2007) USA [43]	Cross-sectional	1045	6 months–6 years	(1) Parental TV time rules (2) Parental TV program rules (3) Parental perception TV helps (4) Parental perception TV hurts (5) Constant TV household (TV was on always or most of the time, even when no one was watching) (parent self-administered questionnaire)	Child screen time (parent self-administered questionnaire)	None	(1) Parental TV time rules were not associated with child TV time. (2) Parental TV program rules was associated with less likelihood of falling outside the AAP guidelines for 5-6-year olds (OR 0.33, 95% CI 0.12–0.90), but not for 0–2-year olds and 3-4-year olds. (3) Parental perception TV helps was associated with greater likelihood of falling outside the AAP guidelines for 0–2-year olds and 5-6-year olds, but not for 3-4-year olds. (4) Parental perception TV hurts was not associated with child TV time. (5) Constant TV household was associated with greater likelihood of falling outside the AAP guidelines for 3-4-year olds, but not for 0–2-year olds and 5-6-year olds.	3

Yalçin et al. (2002) Turkey [44]	Cross-sectional	187	3–6	(1) Mother TV viewing time (2) Father TV viewing time (parent self-administered questionnaire)	Child TV viewing time (hours/weekday) (parent self-administered questionnaire)	None	(1) The TV viewing time of children was significantly and positively correlated with that of mother and father (Pearson's *r* = 0.49, *P* < 0.001, *r* = 0.53, *P* < 0.001) (2) With multiple linear regression analysis the TV viewing time of mother and sibling were significant predictors of that of the child (adjusted *R* ^2^ = 0.45, *P* < 0.001, did not provide *β*).	3

AOR: adjusted odds ratio. AAP: American Academy of Pediatrics.

**Table 4:** Association between parental influence and children's PA time and screen time.

Author (year) country reference	Study design	Sample	Age (years)	Parental influence (measurement)	Child PA (measurement)	Adjusted confounders	Main findings	Quality score
Gubbels et al. (2011) Netherlands [45]	Cross-sectional	2026	5	Parenting practices: (1) Restriction of sedentary behaviour (2) Monitoring activity (screen time and PA) (3) Stimulation to be active (parent self-administered questionnaire)	(1) Child PA time (minutes/day) (2) Child screen time (parent self-administered questionnaire)	Child: (1) BMI *z*-score at age 5 (2) Gender (3) Birth weight (4) Activity and eating style Parent: (5) BMI (6) Educational level (7) Employment (8) Country of birth (9) Maternal age	(1) Restriction of sedentary behaviour was related to more screen time (*β* = 0.09, *P* < 0.01) and less PA (*β* = −0.19, *P* < 0.001). (2) Parent monitoring activity was not associated with child PA time and screen time. (3) Stimulation to be active was positively associated with PA (*β* = 0.12, *P* < 0.001) and negatively with screen time (*β* = −0.12, *P* < 0.001).	6

Vandewater et al. (2005) USA [46]	Cross-sectional	838	6 months–6 years	(1) Parental TV time rules (2) Parental TV program rules (3) Parental negative attitude towards TV (4) Parental presence during children's TV use (parent self-administered questionnaire)	(1) Child TV time (minutes/day) (2) Child frequency of TV viewing (3) Child playing outdoor (parent self-administered questionnaire)	Child: (1) Age (2) Gender Family: (3) Household income (4) Family structure (5) Parent's education (6) Parent's minority status	(1) Parental TV time rule was negatively associated with child TV time (*β* = −0.18, *P* < 0.001). (2) Parental TV program rules did not predict less TV viewing by children (children whose parents have program rules watched 32.5 more minutes/day TV, *P* < 0.001). The relationship between program rules and child TV viewing was mediated by parental presence during viewing. (3) Children of parents with program rules tended to spend more time playing outdoors (mean difference of minutes playing outdoors = 17.7, *P* < 0.1). (4) Parental negative attitude towards TV was directly associated with child TV time (*β* = −0.09, *P* < 0.05) and was mediated by parental TV time rules. (5) Presence during children's TV use was directly associated with child TV time (*β* = 0.29, *P* < 0.001). (6) Children of parents with time rules and program rules tended to watch TV more frequently (mean difference of frequency watching TV were 0.28, *P* < 0.0001, and 1.07, *P* < 0.001, respectively).	4

Smith et al. (2010) Australia [47]	Cross-sectional	764	1.7–5.6	(1) Parent self-efficacy in influencing child's PA and screen time (parent self-administered questionnaire)	(1) Child PA time (2) Child screen time (parent self-administered questionnaire)	(1) Child age (2) Child gender (3) Region (urban versus rural) (4) Maternal education (5) Household income (6) Barriers	(1) Parent high self-efficacy was associated with low likelihood of not meeting PA guidelines (<3 hrs/day) with AOR 0.36 (95% CI 0.21–0.60). (2) Parent high self-efficacy was associated with low likelihood of exceeding screen time recommendation (≥2 hrs/day) with AOR 0.62 (95% CI 0.43–0.87).	6

Spurrier et al. (2008) Australia [48]	Cross-sectional	280	4-5	(1) Role modelling Mother's frequency of walking >30 mins/day Mother's frequency of organised sport Father's frequency of walking >30 mins/day (2) Rules about screen viewing Frequency that TV is on Rules about TV viewing Parents limit exposure to TV advertising (parent self-administered questionnaire)	(1) Children's outdoor playtime (2) Children's screen time (parent self-administered check list)	No	(1) Parental participation in PA (mother's frequency of walking >30 mins/day and frequency of organised sport) was associated with greater outdoor play in preschool children (*P* = 0.04–0.008). (2) Sedentary activity rules (frequency that TV is on, rules about TV viewing, and parents limit exposure to TV advertising) were associated with greater outdoor play in preschool children (*P* < 0.001). (3) Fewer rules about TV watching were associated with increased sedentary indoor behaviour	3

AOR: adjusted odds ratio.

**Table 5:** Papers reporting an association between parental influences and children's PA.

Parental influences	Association with children's physical activity		
	Positive	Negative	No association
*Parenting practices *			
(1) Parents encourage/support PA	20, 21, 23, 24, 26, 30, 32, 45		25, 27, 28
(2) Parental rules			
Restricting rough games inside	24		
TV viewing rules	47, 48	45	
(3) Parent preference (child do the same activities as older siblings)		24	
*Parent role modelling (parents' PA) *	19, 23, 24, 27, 29, 31, 32, 47		22, 28
*Parent perception *			
(1) Parent perception of importance of child PA	23		32
(2) Parent perceptions of children's physical competence	26, 28		
*Parental self-efficacy in PA *	46		
*Parenting style *			30
Dimensions of parenting style (warmth)	30		

**Table 6:** Papers reporting an association between parental influences and children's screen time.

Parent influences	Association with children's screen time		
	Positive	Negative	No association
*Parenting practices *			
(1) Setting TV rules		47	
TV time rules	45	33, 34, 35, 48	38, 43
TV program rules	48	43	
(2) Coviewing TV with child	35, 48		
(3) Monitoring child screen time			45
(4) Meals with TV on	34, 43		
(5) Stimulation to be active		45	
*Parent role modelling (Parent screen time) *	33, 34, 35, 37, 38, 39, 40, 42, 44		
*Parent perception *			
(1) Perception that children spend too much screen time	33, 38		
(2) Parent perception TV helps	37, 43		
(3) Parent perception TV hurts		48	43
*Parental self-efficacy in reducing child screen time *		36, 37, 38, 46	
*Parenting style (authoritative) *		41	

## Strengths and Limitations of This Review

One of the strengths of this review is that we used comprehensive and systematic inclusion and exclusion criteria and a modified quality assessment tool for the critical appraisals of papers. With this quality assessment tool, the strength of evidence from individual study could be assessed based on a quality assessment score. In the present review, the concept of parental influences included not only parents' role modelling and parenting practices that were commonly included in previous reviews, but also general parenting style, parental self-efficacy, and parental perception of PA and screen time, which are important aspects of parenting. In addition, this review searched six databases which enable a wider range of studies to be found.

However, due to the heterogeneity of studies included in the review we were not able to conduct a meta-analysis. Evidence derived from this review was limited by various measurements used regarding parents' and children's PA as well as aspects of parental influences. The definition of children's screen time was inconsistent in the papers reviewed. Study sample sizes in these papers reviewed noticeably varied from 69 to 10,694 where small sample size undermines the study findings. In addition, most studies were cross-sectional which means it is difficult to make causal inference. Therefore, consideration must be given when interpreting the results.
